# Prediction of Cardiovascular Disease Risk among Low-Income Urban Dwellers in Metropolitan Kuala Lumpur, Malaysia

**DOI:** 10.1155/2015/516984

**Published:** 2015-03-02

**Authors:** Tin Tin Su, Mohammadreza Amiri, Farizah Mohd Hairi, Nithiah Thangiah, Awang Bulgiba, Hazreen Abdul Majid

**Affiliations:** ^1^Centre for Population Health (CePH), Department of Social and Preventive Medicine, Faculty of Medicine, University of Malaya, 50603 Kuala Lumpur, Malaysia; ^2^Department of Development Studies, Faculty of Economics and Administration, University of Malaya, 50603 Kuala Lumpur, Malaysia; ^3^Julius Centre University of Malaya (JCUM), Department of Social and Preventive Medicine, Faculty of Medicine, University of Malaya, 50603 Kuala Lumpur, Malaysia

## Abstract

We aimed to predict the ten-year cardiovascular disease (CVD) risk among low-income urban dwellers of metropolitan Malaysia. Participants were selected from a cross-sectional survey conducted in Kuala Lumpur. To assess the 10-year CVD risk, we employed the Framingham risk scoring (FRS) models. Significant determinants of the ten-year CVD risk were identified using General Linear Model (GLM). Altogether 882 adults (≥30 years old with no CVD history) were randomly selected. The classic FRS model (figures in parentheses are from the modified model) revealed that 20.5% (21.8%) and 38.46% (38.9%) of respondents were at high and moderate risk of CVD. The GLM models identified the importance of education, occupation, and marital status in predicting the future CVD risk. Our study indicated that one out of five low-income urban dwellers has high chance of having CVD within ten years. Health care expenditure, other illness related costs and loss of productivity due to CVD would worsen the current situation of low-income urban population. As such, the public health professionals and policy makers should establish substantial effort to formulate the public health policy and community-based intervention to minimize the upcoming possible high mortality and morbidity due to CVD among the low-income urban dwellers.

## 1. Background

Increasing trends in prevalence and number of deaths from cardiovascular diseases (CVD) in developing nations have raised global attentions towards the prevention and control of CVD among these countries [1–7]. Residents of lower socioeconomic backgrounds within these countries experience more CVDs and related complication due to difficulty in health care accessibility and low health literacy [1, 8–11]. Moreover, urban dwellers have worse cardiovascular health compared to rural counterparts [12–14].

Malaysia, a multiethnic nation with a developing economy [15] is also known to experience rapid population growth [16] and urbanization [17]. However, this accelerating economic transition in Malaysia has been accompanied by high prevalence of CVD risk factors in recent years [18–20]. In addition, the risk of CVD is forecasted to further increase in the following decades [21, 22]. Hence, it is crucial to predict the future risk of CVD and construct relevant public health policies and interventions.

We predicted the 10-year CVD risk of urban dwellers in metropolitan Kuala Lumpur. We believe that this is the first work to show the 10-year CVD risk in urban areas especially among low-income residents of Malaysia.

## 2. Methods

### 2.1. Study Design and Data Collection

Data for this study were derived from a cross-sectional household survey conducted in the Community Housing Projects of the Lembah Pantai area of Metropolitan Kuala Lumpur in 2012. The Kuala Lumpur City Hall squatter resettlement program developed these Community Housing Projects in the year 2000. These housings were designated to residents who (i) are married and have at least one child; (ii) have household head's monthly salary of not more than MYR 2,000 (about 526 USD in year 2000); and (iii) do not have a property within the circumference of 35 kilometer from the metropolitan Kuala Lumpur.

Following are the design and process of data collection of the mentioned household survey. 833 households were selected from four Community Housing Projects, namely, PPR Kerinchi, PPR Pantai Ria, PPR Seri Cempaka, and PPR Seri Pantai using the simple random sampling method. There were two components in household survey: questionnaire and medical screening. The questionnaire focused on obtaining information on sociodemographic characteristics, illness, and health service utilization of all household members.

All members from the selected households were briefed about the data collection procedure. Participation of the study was voluntary and written consents were taken from all participants for both parts of the project. Altogether 2,360 adults (18 years old and above) from 833 selected households were invited to predefined medical centers for blood sampling and anthropometrical measurements. Professional teams led by a medical doctor conducted all medical evaluations. Before medical screening, participation of the study was reaffirmed again verbally by the medical screening team in order to ensure that the participant signed the inform consent by himself or herself. The University of Malaya Medical Centre Ethic Committee approved the ethic application for this study (approval number: MEC Ref. Number. 890.161).

From invitees, 1,192 (50.5%) participated. There was no age, gender, or ethnic differences between respondents and nonrespondents. The data was collected from February to November 2012.

### 2.2. Sample Size

Sample size was determined by OpenEpi [23]. According to the data from City Hall Kuala Lumpur, 29,562 housing units were built under the Community Housing Projects until April 20, 2010. Based on the assumption that an average of four persons occupied one unit, the population who resided in Community Housing Project in Kuala Lumpur was estimated as 120,000. The prevalence of high predicted CVD risk (>20%) was taken from the previous similar study conducted in a semirural community in Malaysia that used the Framingham Risk Scoring model. The study estimated that 37% of the respondents had high CVD risk in ten years [21]. Furthermore, we assumed the true frequencies of the surveyed population to lie between ±5 percent confidence limits, the power to be 80%, and the confidence interval to be 95%. The calculated sample size was 358.

**Figure 1:**
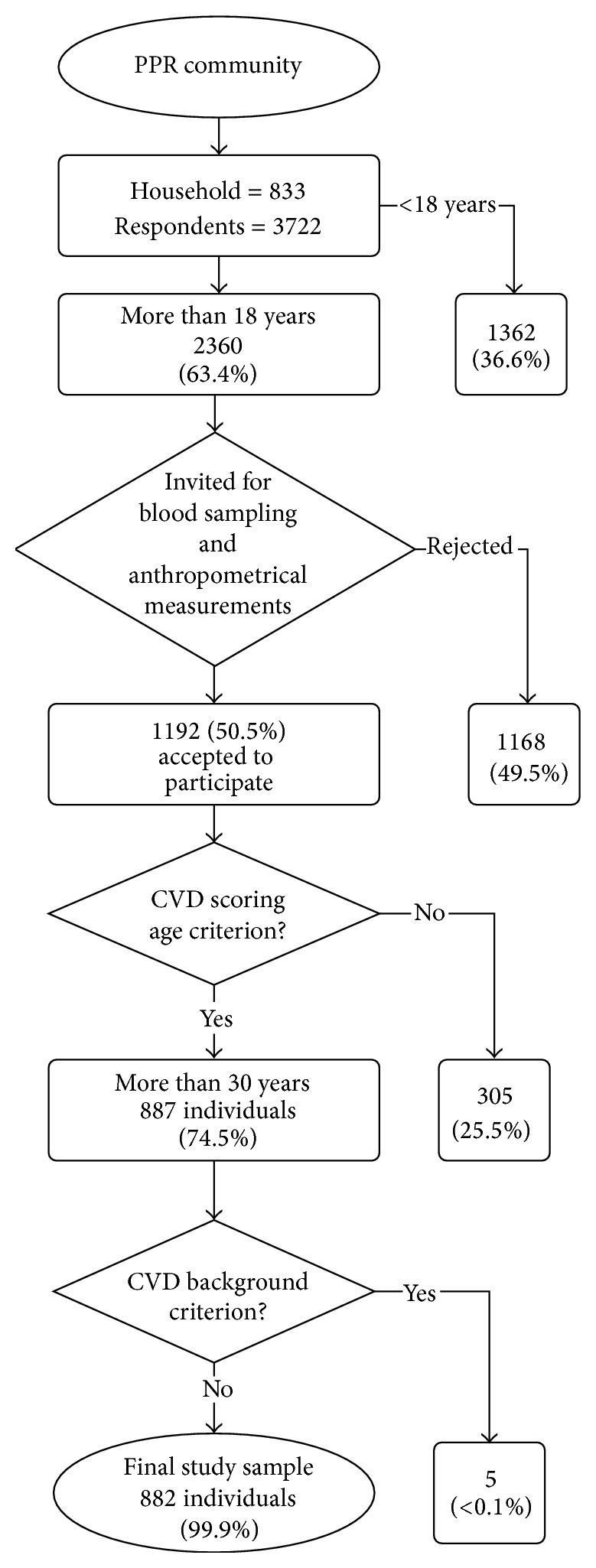
Study sample.

However, we decided to include all eligible participants from the household survey to increase precision of the study findings. We excluded respondents who (i) aged less than 30 years old; and (ii) who had CVD incidence of at least one of the following diseases: coronary death, myocardial infarction, coronary insufficiency, angina, ischemic stroke, haemorrhagic stroke, transient ischemic attack, peripheral artery disease, and heart failure [24, 25]. Subsequently, 882 participants were included for CVD risk prediction model (see Figure 1).

### 2.3. Measurement

Demographic and socioeconomic information, smoking status, antihypertensive medication use, and status of diabetes mellitus were obtained from the questionnaire. The smoking status is positive if the respondent smokes at least one cigarette per day.

Anthropometric measurements included height (measured by SECA 217 Stadiometer for Mobile Height Measurement and rounded to the nearest one mm) and weight (measured by SECA 813 Digital High Capacity Floor Scale and rounded to the nearest 0.1 kg). To obtain body mass index (BMI), the weight was divided by the height in meters squared (kg/m^2^). In the medical screening session, the Arterial blood pressure (measured by Omron HEM 7211 Automatic Blood Pressure Monitor) was evaluated twice in a seated position from the left arm and the mean was considered as the blood pressure status of an individual.

Random blood sugar (RBS) and lipid profile were assessed by Dimension Vista 1500 Intelligent Lab (Siemens, Munich, Germany) System in a certified laboratory of a tertiary hospital. Individuals whose RBS equaled or exceeded 11.0 mmol/L and/or were under diabetic treatment were considered as diabetic [19, 26].

### 2.4. Cardiovascular Disease Risk Scoring

There are two types of sex-specific multivariable risk prediction models. The first model is known as classic Framingham Risk Scoring (FRS) model using laboratory based CVD risk predictors [25]. The model incorporates age (in years), total and high density-lipoprotein (HDL) cholesterol levels, Systolic Blood Pressure (SBP), treatment for hypertension, smoking, and diabetes status.

The FRS has been previously validated in Malaysia by Ng and Chia [22] from the records of 600 patients attending the Family Medicine Clinic of a tertiary hospital. The authors concluded that coronary heart disease (CHD) events predicted by FRS were only marginally higher than observed. And consequently, another study also applied the FRS model to predict CVD risk among semirural population in Malaysia [21]. Moreover, the FRS model has been validated among several populations such as the Chinese [27], Japanese [28, 29], Singaporean, and Korean [28] within Asia. Furthermore, the FRS model can predict the CHD outcomes “fairly well” in South Asian population [30].

D'Agostino et al. [24] proposed the modified version of risk prediction model using nonlaboratory predictors which are routinely available in primary care. These variables included age (in years), body mass index (BMI), Systolic Blood Pressure (SBP), antihypertensive medication use, smoking, and diabetes status.

Previously, both laboratory and nonlaboratory risk prediction models have been compared and studies adopted the notion that both had high agreements in risk characterization [31]. Hence, in this study, we applied both classic and modified methods to predict CVD risk. We used the term lipid-based model and BMI-based model interchangeably for classic laboratory and simplified nonlaboratory based FRS model, respectively.

The steps to predict the ten-year risk of CVD are similar for both models that include (i) summation of FRS points derived from each individual's variables and (ii) conversion of total FRS points into ten-year CVD risk. The detailed method of general cardiovascular risk prediction can be found in D'Agostino et al. [24]. The CVD risk was classified as low (≤6%), moderate (7 to 20%), and high (>20%) [24].

### 2.5. Demographic and Socioeconomic Variables

Age, gender, ethnicity (i.e., Malay, Indian, Chinese, and others), and marital status (i.e., single, married, divorced, and widow/widower) were the sociodemographic variables in our analyses. The socioeconomic status indicators were monthly income adequacy level of less than MYR 1,000 and equal to or more than MYR 1,000 (i.e., we used inflation adjusted cut-off points for poverty line in Malaysia) [32]. Respondent's highest level of education was classified to none (zero years of education), primary (1 to 6 years), secondary (7 to 12 years), and tertiary (above 13 years). Finally, occupational status was broken down to “paid-employee, self-employed, inactive, house maker, and others” subcategories [33, 34]. Inactive subcategory included people who are in retirement and early retirement, who have given up businesses, and who are unemployed. Others included students, part-timer, traineeship, or apprenticeship.

### 2.6. Statistical Analyses

Univariate descriptive analysis showed demographic and socioeconomic characteristics of the sample. The *t*-test (for variables with just two categories) and *F*-test (ANOVA, for variables with more than two categories) results compared the significant difference of predicted CVD risk among categorical variables (prob. < 0.05). Finally, general linear model (GLM) identified the demographic and socioeconomic determinants of the predicted ten-year CVD risk scores. All statistical analyses were computed using the Stata v11.2 (Stata Corp., USA).

## 3. Result

### 3.1. Descriptive Analysis

A total of 882 respondents fulfilled the criteria of CVD risk scoring, that is, being over 30 years old and with no previous CVD incidence.  Table 1 illustrated the characteristics of the sample indicating that the majority of respondents were Malays (81.97%) and married (76.8%).

The mean predicted CVD risks were 11.39 (95% CI; 10.76–12.02) and 11.34 (95% CI; 10.71–11.98) for BMI and lipid-based models, respectively. The median predicted CVD risk for the BMI- and lipid-based models was 8.00 (IQR: 3.90–15.70) and 7.90 (IQR: 3.90–18.40), respectively. There were no significant differences between both models. In addition, according to the lipid-based scoring method (*figures in parentheses indicate BMI-based scoring results*), 20.5% (21.8%) and 38.5% (38.9%) of the respondents were at high and moderate risks of CVD, respectively.

The demographic and socioeconomic differences of predicted ten-year CVD risk were analyzed and presented in Table 2. The results identified that there were significant differences among all variables except in ethnic/race groups. The mean 10-year risk of CVD increases by age and was higher among males and widow/widowers. The respondents with no education and who earned lesser than MYR 1,000 forecasted higher level of 10-year CVD risk. Finally, the inactive respondents had the highest mean predicted in regard to CVD risk compared to other occupational status categories.

**Figure 2:**
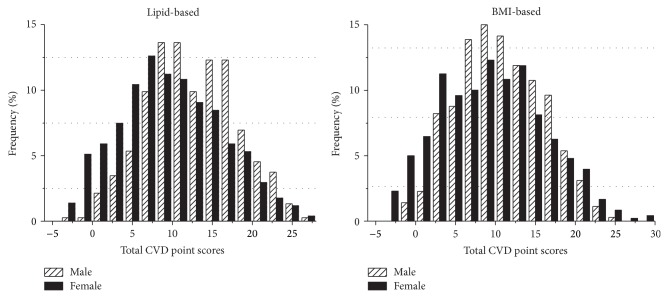
Total predicted cardiovascular disease point scores by gender.

Figure 2 plotted the frequency percentage of the total predicted CVD point scores in male and female strata. In the lipid-based risk score histogram, it is obvious that male participants recorded higher total CVD risk scores compared to female participants in more than 7.5 total CVD risk points; that is, male participants' frequency bars were consistently higher after that point. However, the BMI-based model varied significantly. With a cut-off point of 20 risk scores, the female participants abruptly overtook the male participants and as such the numbers of women who had high CVD risk score were recorded to be higher than the male participants. Furthermore, there was significant difference between both male and female participants in CVD risk scores (*P* < 0.001).

**Figure 3:**
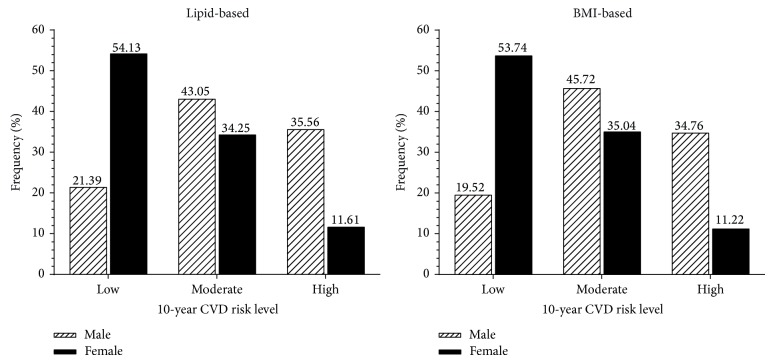
Gender differences in predicted ten-year cardiovascular disease risk levels.

Next, the Chi square result (*P* < 0.001) revealed that the predicted CVD risk was worse in male participants than the female participants. For instance, male participants recorded a high risk of 35.6%, a threefold increase compared to female participants predicted ten-year CVD risk (11.6%). Similarly, the moderate risk of male participants was higher, 43.0%, while female participants' recorded 34.2% (see Figure 3).

### 3.2. Regression Analysis

The General Linear Model (GLM) are depicted in Table 3. Results from both models illustrated that education level, occupation, and marital status were the most significant predictors of the future CVD risk.

The lower levels of education coincide with higher future risks of CVD. For instance, the 10-year CVD risk points of none and primary education levels were the highest compared to tertiary level. Among different occupational categories, inactive and self-employed respondents had the higher 10-year CVD risk compared to the paid-employees. However, house makers tend to have lower predicted CVD risk.

Lastly, the worst 10-year prediction of CVD risk among marital status highlighted married and widow/widower compared to single.

## 4. Discussion

We predicted the burden of ten-year risk of CVD among low-income urban dwellers in Kuala Lumpur and identified the demographic and socioeconomic predictors of the CVD risk. In general, our results tallied with previous findings where older respondents and males were at higher risk of CVD in the next decade [35, 36]. However, in our study we found a contradictory outcome of predicted CVD risk, namely, in different marital statuses. In a previous study, the singles and widows recorded higher CVD risk, contrastingly based on the GLM results we observed that the married and widow/widowers recorded higher predicted CVD risk instead [37]. This could be associated with the cultural variation within the South East Asian region whereby single adults or divorcee lived with family and secured social and emotional protection unlike the western society.

The INTERHEART study, which included 52 countries, concluded that the prevalence and risk of CVDs in urban areas are higher [38], and hence we assumed that 10-year risk of CVD in urban Malaysian would be higher than rural areas. However, our scoring results were lower than the high risk of CVD (>20%) derived from semirural areas; that is, our study revealed 35.6% and 11.6% for men and women, respectively, while semirural areas recorded 55.8% and 15.1% for men and women, respectively [21]. The previous study in semirural areas included higher percentage of elderly respondents and the mean age of the sample participants was 65.4 (SD 8.0) years. The mean age of our study participants was 48.0 (SD 11.7) years. As such the gap of mean age justifies the lower CVD risk predicted in our study unlike previous findings.

In addition, our study underlined that stable employment and job security played a vital role in preventing CVD [39, 40]. Therefore, inactive individuals, self-employed, and unemployed/job seekers faced a higher risk of CVD compared to paid-employees. Generally, paid-employees both in private and governmental sectors occupy full-time positions and have a secured regular income. Hence, occupational status is a significant determinant of CVD risk in low-income urban areas within Kuala Lumpur, Malaysia, too as reflected in several previous studies [39–43]. Therefore, investing in educating individuals, creating more job opportunities, securing stable income, or increasing income level of the economically disadvantaged population is beneficial and highly recommended. These specific efforts may lessen the CVD risk and enhance the health condition among low-income urban dwellers [20, 44, 45].

### 4.1. Strength

Our study has the following strengths. The ten-year CVD risk was the first study in the region and in Malaysia to evaluate the future risk of CVD among low-income urban dwellers. In addition, powerful international methodology was applied to predict CVD risk. The results obtained from both BMI- and lipid-based models are made available for international comparison.

The CVD risk prediction models, which we used to predict the ten-year CVD risk, have the following advantages over other methods. Firstly, our models had advanced scoring method compared to the previous Framingham scoring model [25, 46]. Secondly, unlike the SCORE method (which only predicts CVD risk based on fatal CVD events) [46], FRS considers both fatal and nonfatal events of CVD. Thirdly, the SCORE methods consider the high risk of CVD as >5% whereas it is >20% in FRS, which indicates a more logical classification. Fourthly, the FRS scoring has been widely validated in Asia [22, 27–29] and worldwide in recent years [47–50]. Besides, the FRS is the only risk prediction model which has been validated in Malaysia previously [22]. Lastly, FRS includes ages up to 75, whereas other models only cover until the age of 65 [51–53].

### 4.2. Limitation

Our study identified few limitations. Firstly, we included the low-income adults particularly from Kuala Lumpur. As such, our study may not represent the whole low-income urban population of Malaysia. Hence, a geographically diverse representative study would fill this gap of prediction of CVD risk in urban areas of Malaysia. Secondly, obtaining the fasting blood samples to diagnose diabetes mellitus may result in greater precision in predicting CVD risk. However, we used different scoring methods based on BMI and lipid in order to minimize this effect.

## 5. Conclusion

Our study revealed that one out of five low-income urban dwellers stands a high chance of having CVD within 10 years. In addition, our study observed socioeconomic inequality in future incidence of CVD. The uneducated and unstable job status clearly had high predicted risk of CVD rates in upcoming decade. Health care expenditure, other illness related costs, and loss of productivity due to CVD would further worsen the current situation of low-income urban population and hamper the financial sustainability of a highly government subsidized health care system.

The public health professionals and policy makers should establish substantial efforts to formulate the public health policy and community based intervention in order to curb the upcoming possible high mortality and morbidity due to CVD among low-income urban dwellers.

## Figures and Tables

**Table 1:** Characteristics of the sample (*n* = 882).

	*N* (%)
Age	
30 to 39	228 (22.85)
40 to 49	287 (32.54)
50 to 59	209 (23.70)
≥60	158 (17.91)
Gender	
Male	374 (42.40)
Female	508 (57.60)
Ethnicity	
Malay	723 (81.97)
Indian	144 (16.33)
Chinese/others	15 (1.70)
Marital status	
Married	659 (76.81)
Divorced	71 (8.28)
Widow/widower	55 (6.41)
Single	73 (8.51)
Education	
None	70 (8.16)
Primary	195 (22.73)
Secondary	551 (64.22)
Tertiary	42 (4.90)
Income	
MYR^1^ < 1,000	168 (19.58)
MYR ≥ 1,000	690 (80.42)
Occupation	
Paid-employee	332 (38.69)
Self-employed	131 (15.27)
House maker	223 (25.99)
Others	126 (14.69)
Inactive	46 (5.36)

^1^Malaysian Ringgit (1 USD ≈ 3.1 MYR in 2012).

**Table 2:** The average predicted ten-year cardiovascular disease risk (*n* = 882).

	BMI-based CVD risk Mean (95% CI^1^)	Lipid-based CVD risk Mean (95% CI)
Age		
30 to 39	3.40 (3.03–3.78)	3.77 (3.31–4.24)
40 to 49	8.50 (7.80–9.21)	9.01 (8.16–9.86)
50 to 59	15.83 (14.63–17.02)	15.50 (14.25–16.76)
≥60	22.29 (20.97–23.61)	21.00 (19.62–22.38)
*F*-test (prob.)	294.38 (*P* < 0.0001)	201.08 (*P* < 0.0001)
Gender		
Male	15.74 (14.75–16.72)	15.59 (14.59–16.60)
Female	8.19 (7.50–8.88)	8.21 (7.51–8.91)
*t*-test (prob.)	12.67 (*P* < 0.0001)	12.21 (*P* < 0.0001)
Ethnicity		
Malay	11.24 (10.56–11.93)	11.09 (10.41–11.78)
Indian	11.69 (10.02–13.36)	12.36 (10.62–14.10)
Chinese/others	15.51 (9.46–21.56)	13.62 (7.85–19.39)
*F*-test (prob.)	1.57 (*P* > 0.1)	1.48 (*P* > 0.1)
Marital status		
Single	8.76 (6.86–10.67)	8.29 (6.47–10.11)
Married	11.41 (10.68–12.14)	11.04 (10.32–11.76)
Divorced	12.50 (10.31–14.69)	11.90 (9.64–14.15)
Widow/widower	14.73 (11.90–17.65)	13.61 (10.82–16.41)
*F*-test (prob.)	4.41 (*P* < 0.01)	3.71 (*P* < 0.05)
Education		
None	16.43 (13.89–18.98)	15.65 (13.14–18.16)
Primary	15.29 (13.85–16.72)	14.25 (12.83–15.68)
Secondary	9.93 (9.20–10.65)	9.70 (8.98–10.42)
Tertiary	6.10 (3.88–8.31)	6.00 (3.82–8.19)
*F*-test (prob.)	28.27 (*P* < 0.0001)	22.67 (*P* < 0.0001)
Income		
MYR^2^ < 1,000	13.26 (11.65–14.87)	12.52 (10.96–14.08)
MYR ≥ 1,000	11.05 (10.36–11.75)	10.68 (10.00–11.36)
*t*-test (prob.)	2.69 (*P* < 0.01)	2.28 (*P* < 0.05)
Occupation		
Paid-employee	9.37 (8.48–10.25)	9.16 (8.28–10.04)
Self-employed	13.53 (11.83–15.23)	13.44 (11.68–15.21)
Inactive	22.36 (19.86–24.85)	21.65 (19.18–24.12)
House maker	7.99 (6.97–9.02)	7.40 (6.49–8.31)
Others	17.16 (15.37–18.95)	16.07 (14.28–17.86)
*F*-test (prob.)	47.31 (*P* < 0.0001)	45.53 (*P* < 0.0001)

^1^Confidence Interval; ^2^Malaysian Ringgit (1 USD ≈ 3.1 MYR in 2012).

**Table 3:** Determinants of the predicted ten-year cardiovascular disease risk (*n* = 882).

	GLM based on BMI (Coef.)	GLM based on Lipid (Coef.)
Ethnicity		
Malay	0.17 (−1.36–1.71)	0.15 (−1.37–1.68)
Education		
None	7.47^***^ (3.99–10.95)	6.85^***^ (3.40–10.31)
Primary	7.46^***^ (4.53–10.39)	6.82^***^ (3.91–9.72)
Secondary	3.27^*^ (0.60–5.95)	3.25^*^ (0.60–5.91)
Income		
MYR^1^ ≥ 1000	−0.78 (−2.26–0.68)	−0.54 (−2.01–0.92)
Occupation		
Inactive	8.17^***^ (6.44–9.90)	7.59^***^ (5.87–9.30)
House maker	−2.47^***^ (−3.94–−1.00)	−2.74^***^ (−4.20–−1.29)
Others	8.25^*^ (1.31–15.20)	7.50^*^ (0.61–14.39)
Self-employed	3.35^***^ (1.61–5.08)	3.53^***^ (1.81–5.26)
Marital status		
Married	3.92^***^ (1.78–6.06)	3.94^***^ (1.82–6.06)
Divorced	1.49 (−1.35–4.35)	1.57 (−1.26–4.40)
Widow/widower	3.59^*^ (0.50–6.67)	3.38^*^ (0.32–6.45)
Constant	2.82 (−0.86–6.51)	2.54 (−1.11–6.20)

Note 1: non-Malay (ethnicity), tertiary level (education), MYR <1000 (income), paid-employee (occupation), and single (marital status) subcategories were excluded from the dummy variables as reference categories.

Note 2: ^*^ *P* < 0.05, ^***^ *P* < 0.001.

^1^Malaysian Ringgit.
